# Preoperative Prediction of Long-Term Survival After Surgery in Patients with Resectable Pancreatic Ductal Adenocarcinoma

**DOI:** 10.1245/s10434-024-15648-4

**Published:** 2024-06-26

**Authors:** Takanori Konishi, Shigetsugu Takano, Tsukasa Takayashiki, Daisuke Suzuki, Nozomu Sakai, Isamu Hosokawa, Takashi Mishima, Hitoe Nishino, Kensuke Suzuki, Shinichiro Nakada, Masayuki Ohtsuka

**Affiliations:** Department of General Surgery, Chiba University Graduate School of Medicine, Chiba, Japan

**Keywords:** Resectable pancreatic ductal carcinoma, Prognostic factor, Upfront surgery, Neoadjuvant therapy, Span-1, PNI, LMR, Long-term survival

## Abstract

**Background:**

Although some clinical trials have demonstrated the benefits of neoadjuvant therapy for resectable pancreatic ductal adenocarcinoma (PDAC), its optimal candidate has not been clarified. This study aimed to detect predictive prognostic factors for resectable PDAC patients who underwent upfront surgery and identify patient cohorts with long-term survival without neoadjuvant therapy.

**Patients and Methods:**

A total of 232 patients with resectable PDAC who underwent upfront surgery between January 2008 and December 2019 were evaluated.

**Results:**

The median overall survival (OS) time and 5-year OS rate of resectable PDAC with upfront surgery was 31.5 months and 33.3%, respectively. Multivariate analyses identified tumor diameter in computed tomography (CT) ≤ 19 mm [hazard ratio (HR) 0.40, *p *< 0.001], span-1 within the normal range (HR 0.54, *p *= 0.023), prognostic nutritional index (PNI) ≥ 44.31 (HR 0.51, *p *< 0.001), and lymphocyte-to-monocyte ratio (LMR) ≥ 3.79 (HR 0.51, *p *< 0.001) as prognostic factors that influence favorable prognoses after upfront surgery. According to the prognostic prediction model based on these four factors, patients with four favorable prognostic factors had a better prognosis with a 5-year OS rate of 82.4% compared to others (*p *< 0.001). These patients had a high R0 resection rate and a low frequency of tumor recurrence after upfront surgery.

**Conclusions:**

We identified patients with long-term survival after upfront surgery by prognostic prediction model consisting of tumor diameter in CT, span-1, PNI, and LMR. Evaluation of anatomical, biological, nutritional, and inflammatory factors may be valuable to introduce an optimal treatment strategy for resectable PDAC.

**Supplementary Information:**

The online version contains supplementary material available at 10.1245/s10434-024-15648-4.

Pancreatic ductal adenocarcinoma (PDAC) is one of the most lethal malignancies in the world.^1^ Surgical resection is the only potentially curative treatment for PDAC;^2^ however, the prognosis after surgery is poor due to the aggressive biological behavior of PDAC. Multidisciplinary treatment has been developed for PDAC, and adjuvant chemotherapy after surgery has become a standard treatment option for PDAC according to the results of previous clinical trials.^3,4^ Neoadjuvant therapy has also been reported to improve the patient’s prognosis for borderline resectable PDAC.^5,6^ For resectable PDAC, the efficacy of neoadjuvant therapy is still controversial.^7–9^ Recently, some studies demonstrated the survival benefits of neoadjuvant chemotherapy even in patients with resectable PDAC.^10,11^ Because neoadjuvant therapy has drawbacks, such as adverse events, which can influence the following planned operation,^12–14^ the indication of neoadjuvant therapy for resectable PDAC must be well defined. A previous study suggested that the effect of neoadjuvant therapy on prolonging survival could be minimal in early stage PDAC.^15–17^ Therefore, preoperative factors predicting favorable oncological outcomes can facilitate the selection of appropriate treatment for patients with resectable PDAC.

Previous reports have shown that several clinicopathological factors related to anatomical factors of the primary tumor (such as location, size, and venous invasion), tumor markers [such as carbohydrate antigen 19-9 (CA19-9), pancreatic cancer-associated antigen (DUPAN-2), and s-pancreas-1 antigen (span-1)], nutritional parameters [such as prognostic nutritional index (PNI) and controlling nutritional status score], and inflammatory markers [such as lymphocyte-to-monocyte ratio (LMR) and neutrophil-to-lymphocyte ratio (NLR)] were associated with patient survival after PDAC surgery.^18,19^ Although these factors have been identified as independent prognostic factors, no single marker is sufficient to optimally predict the survival of individual patients and to be practically used for clinical decision making. We hypothesized that combining multiple prognostic factors may improve the quality of clinical outcome prediction after surgery. Since neoadjuvant chemotherapy is not theoretically necessary for PDAC patients who can be cured by upfront surgery with or without adjuvant therapy, we focused on the preoperative prognostic factors of the patients who underwent upfront surgery. In this study, we sought to examine preoperative prognostic factors that reflect a favorable prognosis of resectable PDAC to identify the patients who have long-term survival only by upfront surgery.

## Patients and Methods

### Patients

We retrospectively analyzed the medical records of 232 consecutive patients with resectable PDAC who underwent upfront surgery at the Department of General Surgery at Chiba University (Chiba, Japan) between January 2008 and December 2019. Prior to the operation, all patients underwent dynamic multidetector-row computed tomography, endoscopic ultrasound, magnetic resonance imaging, and positron emission tomography to evaluate the resectability status. Resectable PDAC was defined to fulfill the following criteria: (1) no contact or invasion with the celiac artery, common hepatic artery, or superior mesenteric artery; (2) no contact or contact less than 180° without occlusion to the superior mesenteric vein or portal vein; and (3) no distant metastases, as shown in the NCCN guideline for pancreatic adenocarcinoma version 2 (2021). Written informed consent was obtained from all patients according to the ethical standards of the Declaration of Helsinki (1975). The study protocol was approved by the Committee on Human Research of Chiba University School of Medicine (approval code: #3302).

### Preoperative Parameters

All patients underwent blood tests before surgery to evaluate preoperative parameters such as tumor markers, nutritional parameters, and inflammatory markers. If the patients had jaundice or cholangitis because of tumor invasion of the bile duct, the preoperative patient parameters were evaluated after biliary drainage. The PNI was calculated using the following formula: 10 × serum albumin (g/dl) + 0.005 × absolute peripheral lymphocyte counts (/mm^3^). The LMR is defined as the ratio of absolute peripheral lymphocyte and monocyte counts. The NLR is defined as the ratio of absolute peripheral neutrophil and lymphocyte counts.

### Operation

We performed pancreaticoduodenectomy, distal pancreatectomy, or total pancreatectomy depending on the tumor location as previously reported.^20^ Regional lymphadenectomy and para-aortic lymph node sampling were performed. Combined portal vein resection and reconstruction were performed when portal vein invasion was suspected during surgery. The nerve plexus around the common hepatic artery and the superior mesenteric artery were not dissected for resectable PDAC. The pancreatic dissection margin was evaluated by frozen-section analyses during surgery, and additional pancreatic resection was performed to obtain a negative resection margin.

### Pathological Evaluation

Pathological specimens were examined based on the TNM classification of Malignant Tumors 8th edition of the Union for International Cancer Control. Lymphatic, venous, and nerve invasions were assessed according to the Classification of the Pancreatic Carcinoma from the Japan Pancreas Society.^21^ The status of the resection margin was microscopically evaluated. A positive resection margin (R1) is defined as a tumor with the presence of cancer cells at the transection line.

### Adjuvant Chemotherapy and Follow-up

The patients underwent routine adjuvant chemotherapy for 6 months once they were deemed medically fit for it. Adjuvant chemotherapy regimens followed were gemcitabine (GEM) monotherapy, S-1 monotherapy, or combination therapy of GEM and S-1. Regarding follow-up, patients underwent blood tests, including tumor markers, every 3 months, and dynamic multidetector-row computed tomography every 6 months.

### Statistical Analyses

Disease-free survival (DFS) and overall survival (OS) were analyzed using the Kaplan–Meier method, and statistical significance was examined using the log-rank test. Multivariate analysis was performed using the Cox proportional hazards model. Additionally, baseline variables with *p *< 0.05 on the univariate analysis were included in the multivariate analysis. The association between groups stratified by prognostic prediction model and clinicopathological features was assessed using the Chi-squared test, Fisher’s exact test, or *t*-test. All tests were two-tailed, and *p *< 0.05 denoted a statistically significant difference. Data were analyzed using JMP software (SAS Institute, Cary, NC, USA).

## Results

### Patients

**Fig. 1:**
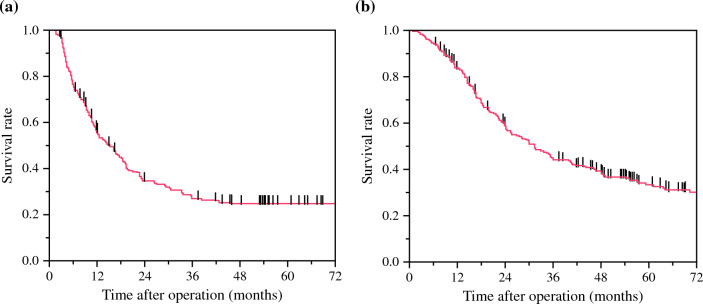
**a** Disease-free survival rate after upfront surgery in patients with resectable pancreatic ductal adenocarcinoma (PDAC). **b** Overall survival rate after upfront surgery in patients with resectable PDAC

The patient characteristics are illustrated in Table 1. Among the 232 patients with resectable PDAC, 147 patients had pancreatic head cancer and 85 patients had pancreatic body cancer or pancreatic tail cancer. Two hundred twenty-three patients underwent pancreatectomy with R0 or R1 resection, while nine patients had only laparotomy due to distant metastases or peritoneal dissemination detected during the operation. Fifty-four patients underwent combined portal vein resection and R0 resection was conducted in 73.7% of the patients with pancreatectomy. The completion rate of adjuvant chemotherapy for 6 months was 55.2%. We analyzed the prognosis of patients with resectable PDAC after upfront surgery using the Kaplan–Meier method. The DFS curve showed that the 5-year DFS rate was 24.9% (Fig. 1a), while the OS curve showed that the median survival time was 31.5 months and the 5-year OS rate was 33.3% (Fig. 1b). These results suggested that many patients experienced tumor recurrence after surgery leading to poor prognosis, but there was a patient cohort with long-term survival after upfront surgery.

**Table 1:** Characteristics of patients with resectable pancreatic ductal adenocarcinoma

Parameter	*n *= 232
Age (years)	70.5 (31–89)
Gender (male/female)	135/97
Tumor location (Ph/Pbt)	147/85
Diameter of the tumor in CT (mm)	22 (0–60)
CA19-9 level (U/ml)	134 (0–15,600)
DUPAN-2 level (U/ml)	99 (9.9–1600)
Span-1 level (U/ml)	49 (1–7500)
PNI	46.05 (17.89–57.77)
LMR	4.61 (0.92–12.48)
NLR	2.41 (0.85–15.09)
Operative method (PD/DP/TP)	141/80/2
Portal vein resection (rate)	54 (24.2%)
Operation time (min)	424 (120–825)
Blood loss (ml)	610 (15–5655)
Degree of differentiation (well/mode/poor/others)	72/115/27/9
pT factor (UICC T1/T2/T3/T4)	34/162/27/0
pN factor (UICC N0/N1/N2)	66/94/63
R0 resection (rate)	171 (73.7%)
Completion of adjuvant chemotherapy (rate)	123 (55.2%)

*Ph* pancreatic head, *Pbt* pancreatic body or tail, *PNI* prognostic nutrition index, *LMR* lymphocyte-to monocyte ratio, *NLR* neutrophil-to-lymphocyte ratio, *PD* pancreaticoduodenectomy, *DP* distal pancreatectomy, *TP* total pancreatectomy

### Prognostic Factors for Favorable Overall Survival after Upfront Surgery

In the era of multidisciplinary therapy for PDAC, prediction of the oncological outcome by upfront surgery at the time of diagnosis may be valuable to select optimal candidates for neoadjuvant therapy. Previous reports have demonstrated that anatomical factors, biological factors, nutritional parameters, and inflammatory markers were associated with patient survival.^18,19^ Therefore, we sought to evaluate the diameter of tumor in preoperative computed tomography (CT), preoperative tumor markers (CA19-9, DUPAN-2, and span-1), preoperative PNI, preoperative LMR, and preoperative NLR as prognostic factors for predicting favorable outcomes after upfront surgery. Since there was no normal range demonstrated regarding PNI, LMR, and NLR, we determined the cutoff values of PNI, LMR, and NLR, as well as tumor diameter in CT, to predict long-term survival. The receiver operating characteristic analysis of these factors associated with 5-year OS revealed that the optimal cutoff value of tumor diameter in CT, PNI, LMR, and NLR were 19 mm, 44.31, 3.79, and 2.62, respectively (Supplementary Table 1). On the other hand, upper normal limits of CA19-9 (≤ 37.0 U/ml), DUPAN-2 (≤ 150 U/ml), and span-1 (≤ 30 U/ml) in our institution were used as cutoff values of these tumor markers in this study.

We then performed univariate and multivariate analyses to investigate the preoperative factors that affect OS after surgery (Table 2). Univariate analyses showed that tumor diameter on CT ≤ 19 mm (*p *< 0.001), CA19-9 level within normal range (*p *= 0.040), DUPAN-2 level within normal range (*p *< 0.001), span-1 level within normal range (*p *< 0.001), PNI ≥ 44.31 (*p *< 0.001), LMR ≥ 3.79 (*p *< 0.001), and NLR ≤ 2.62 (*p *= 0.006) were associated with OS after surgery. Furthermore, multivariate analyses demonstrated that tumor diameter on CT [hazard ratio (HR) 0.40; 95% confidence interval (95% CI) 0.26–0.60; *p *< 0.001], span-1 level (HR 0.54; 95% CI 0.32–0.92; *p *= 0.023), PNI (HR 0.51; 95% CI 0.33–0.75; *p *< 0.001), and LMR (HR 0.51; 95% CI 0.36–0.75; *p *< 0.001) were identified as independent risk factors that affect OS.

**Table 2:** Univariate and multivariate analyses of predictive factors for overall survival

Factors		Univariate analysis			Multivariate analysis		
		Hazard ratio	95% CI	*p*-value	Hazard ratio	95% CI	*p*-value
Age (years)	< 60	1.06	0.66–1.72	0.81			
	60 ≤						
Gender	Male	1.07	0.77–1.48	0.69			
	Female						
Tumor location	Body/tail	0.81	0.58–1.13	0.21			
	Head						
Diameter of the tumor in CT (mm)	≤ 19	0.35	0.24–0.52	< 0.001	0.40	0.26–0.60	< 0.001
	19 <						
CA19-9 level (U/ml)	≤ 37.0	0.66	0.45–0.98	0.040	0.64	0.38–1.08	0.093
	37.0 <						
DUPAN-2 level (U/ml)	≤ 150	0.57	0.41–0.79	< 0.001	1.13	0.75–1.70	0.55
	150 <						
Span-1 level (U/ml)	≤ 30	0.50	0.35–0.71	< 0.001	0.54	0.32–0.92	0.023
	30 <						
PNI	44.31 ≤	0.44	0.31–0.61	< 0.001	0.51	0.33–0.75	< 0.001
	< 44.31						
LMR	3.79 ≤	0.44	0.31–0.61	< 0.001	0.51	0.36–0.75	< 0.001
	< 3.79						
NLR	≤ 2.62	0.63	0.45–0.88	0.006	1.18	0.80–1.75	0.40
	2.62 <						

*PNI* prognostic nutrition index, *LMR* lymphocyte-to monocyte ratio, *NLR* neutrophil-to-lymphocyte ratio

### Prediction of Oncological Outcomes after Upfront Surgery for Resectable Pancreatic Ductal Adenocarcinoma

**Fig. 2:**
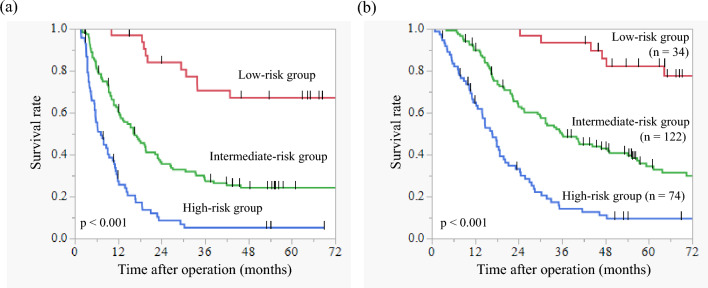
**a** Disease-free survival rate after upfront surgery according to the prognostic prediction model in patients with resectable pancreatic ductal adenocarcinoma (PDAC). **b** Overall survival rate after upfront surgery according to the prognostic prediction model in patients with resectable PDAC. Low-risk group, *n *= 34; intermediate-risk group, *n *= 122; high-risk group, *n *= 74

Our data so far suggested that tumor diameter, span-1, PNI, and LMR before treatment can predict survival after surgery for resectable PDAC. Kaplan–Meier survival analyses showed that the 5-year OS rates of patients with tumor diameter in CT ≤ 19 mm, those with span-1 level within normal range, those with PNI ≥ 44.31, and those with LMR ≥ 3.79 were 55.4%, 47.5%, 42.4%, 42.7%, respectively (Supplementary Fig. 1). Next, we sought to clarify whether a combination of these four factors may facilitate the prediction of a favorable prognosis of the patients with resectable PDAC after upfront surgery. Then, we established the prognostic prediction model based on the total number of these four favorable prognostic factors. In this model, patients with scores of 4 points were categorized as low-risk group, patients with scores of 2 or 3 points were categorized as intermediate-risk group, and patients with scores of 0 or 1 points were categorized as high-risk group. The number of patients in the low-risk groups, the intermediate-risk group, and the high-risk group were 34, 122, 74 cases, respectively. Two cases were excluded due to the lack of data regarding the span-1 level. On the Kaplan–Meier survival analyses as shown in Fig. 2, both the DFS rate and the OS rate were significantly stratified by the prognostic prediction model (*p *< 0.001 and *p *< 0.001, respectively). Notably, the 5-year OS rates of cases in the low-risk group were 82.4%. These results indicated that this prognostic prediction model may extract patient cohort with long-term survival even after the upfront surgery. To identify the pathological characteristics of the patients in the low-risk group that were determined by preoperative factors, patients in the low-risk group were compared with other cases, as presented in Table 3. The low-risk group exhibited significantly lower levels of lymphatic, venous, and neural invasion than other cases (*p *= 0.003, *p *< 0.001, and *p *= 0.005, respectively). Patients in the low-risk group also showed a significantly higher frequency of pN0 cases than other cases (*p *= 0.002). These results suggested that PDAC in the low-risk group had minimal invasive and metastatic potential. In addition, the low-risk group tended to achieve higher R0 resection rates than other cases (*p *= 0.06). Then, we evaluated the adjuvant therapy and tumor recurrence after surgery as presented in Table 3. There was no significant difference in the ratio of patients who started to receive adjuvant therapy after surgery between the low-risk group and other cases (*p *= 0.48). However, completion rate of adjuvant therapy for 6 months tended to be higher in the low-risk group than other cases (*p* = 0.10) because the intermediate-risk and high-risk groups had higher discontinued rate due to early recurrence during adjuvant therapy than the low-risk group (*p *< 0.001). During follow-up after surgery, the low-risk group experienced significantly lower frequency of tumor recurrence than other cases (*p *< 0.001). Furthermore, the low-risk group experienced liver metastases and peritoneal dissemination less frequently than other cases, whereas the low-risk group experienced lung metastases more frequently than other cases.

**Table 3:** Clinicopathological characteristics of patients with resectable pancreatic ductal adenocarcinoma in the low-risk group

Factors	Low-risk group (*n *= 34)	Other cases (*n *= 187)	*p*-value
Pathological findings			
Tumor differentiation (well/mod/por/others)	13/16/5/0	58/98/22/9	0.85
pT factor (UICC 8th) (pT1/pT2–4)	16/18	18/169	< 0.001
pN factor (UICC 8th) (pN0/pN1–2)	18/16	48/139	0.002
Lymphatic invasion (ly0,1/ly2,3)	27/7	99/88	0.003
Venous invasion (v0,1/v2,3)	31/3	120/67	< 0.001
Neural invasion (ne0,1/ne2,3)	24/10	83/104	0.005
Resection margin status (R0/R1)	30/4	139/48	0.060
Treatment after operation			
Initiation of adjuvant chemotherapy ( + / − )	25/9	147/40	0.48
Completion of adjuvant chemotherapy ( + / − )	23/11	98/89	0.10
Recurrence of the disease			
Tumor recurrence ( + / − )	11/23	149/38	< 0.001
Recurrence site			0.035
Liver metastases	1 (9.1%)	38 (25.5%)	
Lung metastases	5 (45.5%)	17 (11.4%)	
Peritoneal dissemination	0 (0%)	13 (8.7%)	
Local recurrence	3 (27.3%)	50 (33.6%)	
Remnant pancreas recurrence	1 (9.1%)	2 (1.3%)	
Others	1 (9.1%)	9 (6.0%)	
Multiple organs	0 (0%)	18 (12.1%)	

Next, we confirmed the significance of this prognostic prediction model in the patients who achieved an R0 resection and completed adjuvant therapy. In this subgroup analysis, OS rate was also significantly stratified by the prognostic prediction model [5-year OS rates: low-risk group 88.4% (*n *= 19), intermediate-risk group 50.9% (*n *= 57), high-risk group 17.7% (*n *= 17), *p *< 0.001].

## Discussion

In this study, we identified short tumor diameter in CT, low span-1 level, high PNI, and high LMR as predictive factors of favorable prognosis after upfront surgery for patients with resectable PDAC. Furthermore, we established a prognostic prediction model based on these preoperative factors to identify the patient cohort with long-term survival after surgery. Our prognostic prediction model may be valuable for the decision-making regarding the optimal treatment strategy for patients with resectable PDAC in the era of multidisciplinary treatment for PDAC.

The prognosis of PDAC is far from satisfactory even after curative resection; therefore, multidisciplinary treatment is thought to be important in improving the prognosis of PDAC.^22^ Our data revealed that the median survival time after upfront surgery for resectable PDAC patients was 31.5 months, which was consistent with previous reports.^23^ The effect of neoadjuvant therapy for resectable PDAC remains controversial. While the NORPACT-1 trial did not show a survival benefit of neoadjuvant chemotherapy for resectable PDAC,^9^ some clinical studies, including PREP-02/JSAP05 trial, suggested that neoadjuvant therapy would be a promising treatment option for patients with resectable PDAC.^10,11,24,25^ Neoadjuvant therapy may affect the general condition of patients and increase postoperative complications.^14,26,27^ Neoadjuvant therapy also has the potential risk of tumor growth, leading to missed opportunity for curative resection. Furthermore, the effect of neoadjuvant chemotherapy may differ according to the progression of the disease.^15,28^ Therefore, indications for neoadjuvant therapy among patients with resectable PDAC need to be established. This study revealed that the 5-year survival rate of patients with resectable PDAC after upfront surgery was 33.3%, suggesting that there is a subset of PDAC patients who can experience a long-term survival without neoadjuvant chemotherapy. Therefore, prediction of the prognosis at the time of diagnosis may be important in determining the optimal candidates of neoadjuvant therapy for resectable PDAC. Our prognostic prediction model could extract 14.8% of patients who had good oncological outcomes even after upfront surgery in the cohort.

Although previous studies have revealed prognostic factors affecting early recurrence after surgery,^29–31^ preoperative factors related to long-term survival are not well investigated. Our prognostic prediction model based on tumor size in CT, span-1 level, PNI, and LMR could identify a patient cohort with a good prognosis categorized as the low-risk group. In the low-risk group, the R0 resection rate was 88.2% and the 5-year survival rate was 82.4%. High R0 resection rate in the low-risk group might be attributed to pathologically small tumor with less invasiveness as presented in Table 3. Pathological examination showed that the low-risk group had less lymphovascular invasion, which is related to metastatic potential and survival. In fact, the low-risk group experienced fewer disease recurrences during follow-up. Furthermore, the low-risk group experienced lung metastases rather than liver metastases and peritoneal dissemination. Previous reports demonstrated that patients with lung recurrence showed significantly better survival than those with other distant metastases,^32^ which may be due to the different tumor biology according to the recurrence patterns.^33^ These results suggested that our prognostic prediction model, which can be identified at the time of diagnosis, reflected the pathological and biological characteristics of resectable PDAC. Neoadjuvant therapy for PDAC has been reported to prolong survival by preoperative downstaging of the PDAC to achieve R0 resection and early treatment of micrometastases that are already present before surgery.^34^ Neoadjuvant therapy can also select the optimal patient for operation by excluding patients with rapid growth and distant metastases.^35^ Previous reports also clarified that the benefit of neoadjuvant therapy on the prognosis was observed only in advanced stage PDAC.^15,36^ Based on the high R0 resection rate, low recurrence rate, and recurrence pattern of patients in the low-risk group, the effect of neoadjuvant therapy for the low-risk group might be minimal.

Four independent prognostic factors identified by our multivariate analyses reflected different important aspects related to disease progression. Tumor size is one of the anatomical factors regarding primary tumors. Previous studies have reported that pathological tumor diameter is a strong predictor of prognosis.^37^ Span-1 can reflect the biological characteristics of PDAC as a tumor marker. In this study, span-1 was an independent prognostic factor affecting OS in multivariate analyses. CA19-9 is the most widely studied tumor marker for PDAC;^38,39^ however, CA19-9 has some limitations, such as false-negative results in patients whose Lewis antigens are Le^a−b−^ and false-positive results in patients with obstructive jaundice.^40^ Span-1 may be a much better surrogate marker of the favorable prognosis for patients with resectable PDAC. PNI is a representative marker of nutritional status, whereas LMR is associated with the systemic inflammatory response of the host. The progression of PDAC can worsen the nutrition of the patient and tumor microenvironment with lymphocytes and monocytes can influence oncological outcomes. A previous study showed that the pretreatment clinical stage based on radiological findings sometimes underestimates pathological tumor progression.^41^ The prognostic prediction model in this study demonstrated that not only the locoregional staging of the tumor but also other oncological and conditional factors could improve the quality of prognostic prediction for resectable PDAC. Therefore, evaluating anatomical factors, biological factors, nutritional status, and inflammatory parameters before treatment may be important for deciding the treatment strategy, including preoperative chemotherapy, for patients with resectable PDAC.

There are some limitations in this study. First, this was a retrospective study conducted in a single institution. A prospective study with a large number of patients is required for further validation of our results. Second, patients in this study received GEM monotherapy, S-1 monotherapy, or combination of GEM and S-1 as adjuvant therapy. A previous study demonstrated that adjuvant therapy with a modified FOLFIRINOX led to better survival than GEM.^42^ The validity of our results needs to be investigated in patients who have adjuvant modified FOLFIRINOX for further clinical utility. Third, our prognostic prediction model clearly classified patients with resectable PDAC with respect to their prognosis, but the effect of neoadjuvant therapy on patients has not been demonstrated. The outcome of neoadjuvant therapy on prognosis according to the prognostic prediction model must be further investigated to establish the indications of neoadjuvant therapy on resectable PDAC.

In conclusion, we identified a cohort of patients with favorable oncological outcomes even after upfront surgery by the prognostic prediction model consisting of tumor diameter on CT, span-1 level, PNI, and LMR. Evaluation of anatomical, biological, nutritional, and inflammatory factors may be valuable in introducing an optimal treatment strategy for patients with resectable PDAC.

## Supplementary Information

Below is the link to the electronic supplementary material.

(a) The overall survival (OS) rate after upfront surgery was stratified according to tumor diameter in computed tomography in patients with resectable pancreatic ductal adenocarcinoma (PDAC). (b) The OS rate after upfront surgery was stratified according to the Span-1 level in patients with resectable PDAC. (c) The OS rate after upfront surgery was stratified according to prognostic nutritional index in patients with resectable PDAC. (d) The OS rate after upfront surgery was stratified according to lymphocyte-to-monocyte ratio in patients with resectable PDAC.
Supplementary file1 (DOC 68 kb)

Supplementary file1 (DOC 68 kb)
