# Supplementary material for: Preoperative Prediction of Long-Term Survival After Surgery in Patients with Resectable Pancreatic Ductal Adenocarcinoma
Source: Ann Surg Oncol. 2024 Jun 26;31(10):6992–7000. doi: 10.1245/s10434-024-15648-4 (PMC11413041; doi:10.1245/s10434-024-15648-4)
Supplement: Supplementary file 1 — (a) The overall survival (OS) rate after upfront surgery was stratified according to tumor diameter in computed tomography in patients with resectable pancreatic ductal adenocarcinoma (PDAC). (b) The OS rate after upfront surgery was stratified according to the Span-1 level in patients with resectable PDAC. (c) The OS rate after upfront surgery was stratified according to prognostic nutritional index in patients with resectable PDAC. (d) The OS rate after upfront surgery was stratified according to lymphocyte-to-monocyte ratio in patients with resectable PDAC. Supplementary file1 (DOC 68 kb) [file 10434_2024_15648_MOESM1_ESM.pptx]

## Slide 1
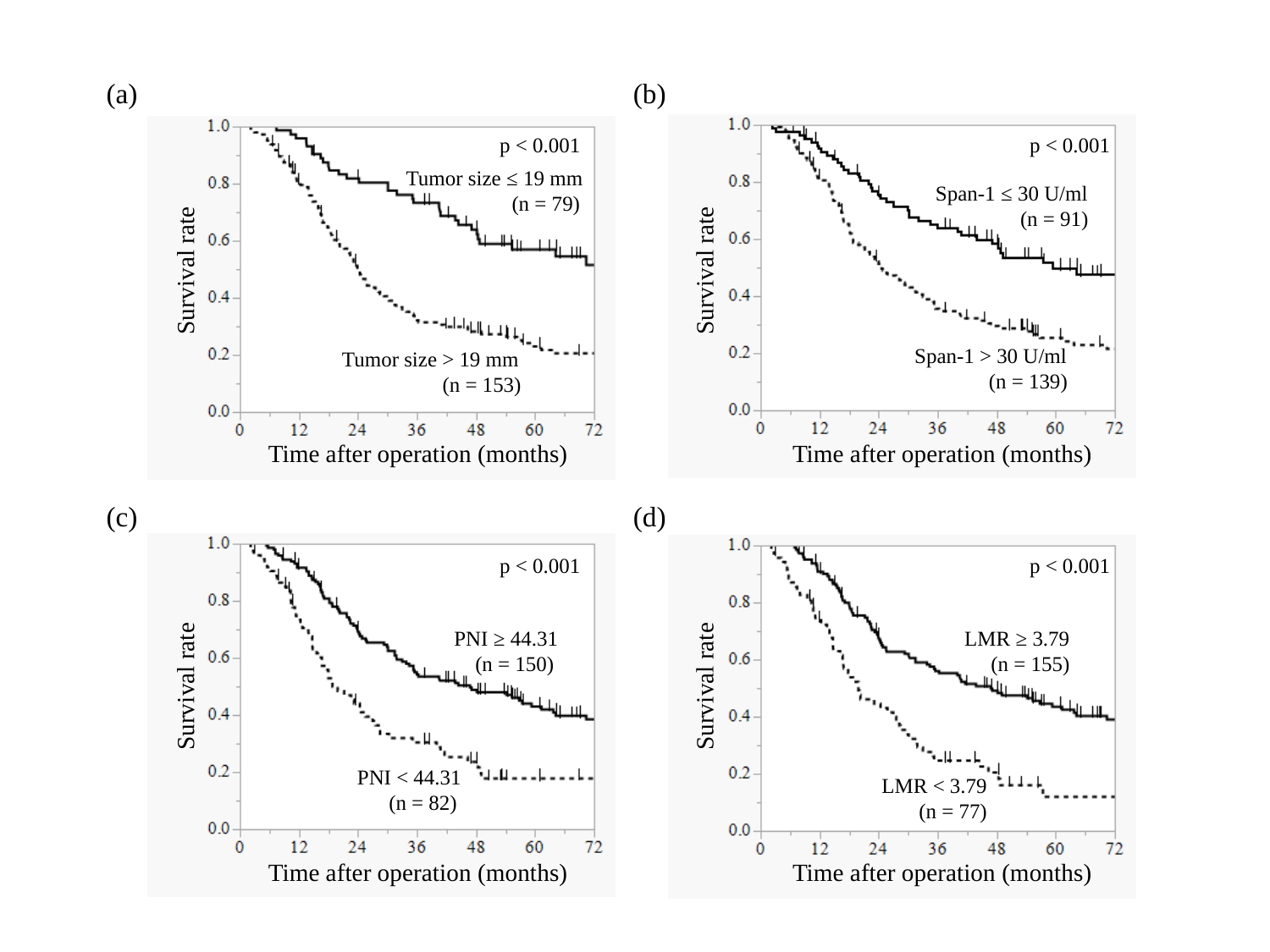

(a)
(b)
p < 0.001
p < 0.001
Tumor size ≤ 19 mm
 (n = 79)
Span-1 ≤ 30 U/ml
 (n = 91)
Survival rate
Survival rate
Span-1 > 30 U/ml
 (n = 139)
Tumor size > 19 mm
 (n = 153)
Time after operation (months)
Time after operation (months)
(c)
(d)
p < 0.001
p < 0.001
PNI ≥ 44.31
 (n = 150)
LMR ≥ 3.79
 (n = 155)
Survival rate
Survival rate
PNI < 44.31
 (n = 82)
LMR < 3.79
 (n = 77)
Time after operation (months)
Time after operation (months)
