# Supplementary material for: Preoperative Prediction of Long-Term Survival After Surgery in Patients with Resectable Pancreatic Ductal Adenocarcinoma
Source: Ann Surg Oncol. 2024 Jun 26;31(10):6992–7000. doi: 10.1245/s10434-024-15648-4 (PMC11413041; doi:10.1245/s10434-024-15648-4)
Supplement: Supplementary file 2 — Supplementary file1 (DOC 68 kb) [file 10434_2024_15648_MOESM2_ESM.doc]

**Supplementary Table 1**

Receiver operating characteristics curve analysis for predicting long-term survival

| Parameter | Cutoff value | ROC | p value |
| --- | --- | --- | --- |
| Diameter of the tumor in CT (mm) | 19 | 0.683 | <0.001 |
| PNI | 44.31 | 0.664 | <0.001 |
| LMR | 3.79 | 0.682 | <0.001 |
| NLR | 2.62 | 0.634 | 0.003 |

PNI: prognostic nutrition index, LMR: lymphocyte-to monocyte ratio, NLR: neutrophil-to-lymphocyte ratio
